# Serotonin signaling to regulate energy metabolism: a gut microbiota perspective

**DOI:** 10.1093/lifemeta/loae039

**Published:** 2024-11-23

**Authors:** Guoli Li, Sijing Dong, Chunhao Liu, Jing Yang, Patrick C N Rensen, Yanan Wang

**Affiliations:** Med-X Institute, Center for Immunological and Metabolic Diseases, First Affiliated Hospital of Xi’an Jiaotong University, Xi’an, Shaanxi 710061, China; Med-X Institute, Center for Immunological and Metabolic Diseases, First Affiliated Hospital of Xi’an Jiaotong University, Xi’an, Shaanxi 710061, China; Department of Endocrinology, First Affiliated Hospital of Xi’an Jiaotong University, Xi’an Jiaotong University, Xi’an, Shaanxi 710061, China; Med-X Institute, Center for Immunological and Metabolic Diseases, First Affiliated Hospital of Xi’an Jiaotong University, Xi’an, Shaanxi 710061, China; Med-X Institute, Center for Immunological and Metabolic Diseases, First Affiliated Hospital of Xi’an Jiaotong University, Xi’an, Shaanxi 710061, China; Department of Endocrinology, First Affiliated Hospital of Xi’an Jiaotong University, Xi’an Jiaotong University, Xi’an, Shaanxi 710061, China; Department of Endocrinology, First Affiliated Hospital of Xi’an Jiaotong University, Xi’an Jiaotong University, Xi’an, Shaanxi 710061, China; Department of Medicine, Division of Endocrinology, and Einthoven Laboratory for Experimental Vascular Medicine, Leiden University Medical Center, Albinusdreef 2, 2333 ZA Leiden, The Netherlands; Med-X Institute, Center for Immunological and Metabolic Diseases, First Affiliated Hospital of Xi’an Jiaotong University, Xi’an, Shaanxi 710061, China; Department of Endocrinology, First Affiliated Hospital of Xi’an Jiaotong University, Xi’an Jiaotong University, Xi’an, Shaanxi 710061, China; Department of Medicine, Division of Endocrinology, and Einthoven Laboratory for Experimental Vascular Medicine, Leiden University Medical Center, Albinusdreef 2, 2333 ZA Leiden, The Netherlands

**Keywords:** serotonin, gut microbiota, metabolism, gut–brain axis

## Abstract

Serotonin is one of the most potent gastrointestinal, peripheral, and neuronal signaling molecules and plays a key role in regulating energy metabolism. Accumulating evidence has shown the complex interplay between gut microbiota and host energy metabolism. In this review, we summarize recent findings on the role of gut microbiota in serotonin metabolism and discuss the complicated mechanisms by which serotonin, working in conjunction with the gut microbiota, affects total body energy metabolism in the host. Gut microbiota affects serotonin synthesis, storage, release, transport, and catabolism. In addition, serotonin plays an indispensable role in regulating host energy homeostasis through organ crosstalk and microbe–host communication, particularly with a wide array of serotonergic effects mediated by diverse serotonin receptors with unique tissue specificity. This fresh perspective will help broaden the understanding of serotonergic signaling in modulating energy metabolism, thus shedding light on the design of innovative serotonin-targeting strategies to treat metabolic diseases.

## Introduction

Serotonin, or 5-hydroxytryptamine (5-HT), is a small monoamine molecule that is widely recognized as a central regulator of neuropsychological processes in human central nervous system (CNS). Moreover, serotonin has crucial physiological functions in the periphery, including modulation of cardiac contraction [1], gut motility and fluid secretion [2], vasoconstriction [3–5], bone formation [6], and coagulation [7]. Throughout phylogenetic history, serotonin plays vital roles in the metabolism of various organisms including unicellular organisms, plants, and animals [8]. A range of serotonergic signaling pathways remain to regulate energy metabolism in primitive animals at different stages of evolution [9–12]. These pathways have been conserved in humans and some primate mammals, functioning both in the CNS and peripheral nervous system.

Over the past few years, the key effects of gut microbiota on the regulation of serotonin level and function have garnered increasing attention. The gut microbiota, often referred to as a “forgotten organ”, is now acknowledged as playing a fundamental role in host physiology, including the regulation of the immune response, synthesis of vitamins, development of the gut barrier, and production of bioactive metabolites [13], as well as regulating dietary energy extraction, fat storage, and insulin sensitivity [14]. Notably, the gut microbiota can influence host serotonin synthesis and serotonergic signaling [15]. Gut-derived serotonin not only impacts the local function of the gut, including motility and secretion, but also communicates with distant organs, such as the liver and pancreas [16]. Furthermore, accumulating evidence has indicated that gut serotonin communicates with the serotonergic signaling in the brain via the gut–brain axis, and *vice versa* [17]. This bidirectional communication highway allows serotonin to play a significant role in host metabolism [18], under the regulatory action of gut microbiota.

In this review, we summarize the present status of the role of gut microbiota in serotonin metabolism, and discuss the various mechanisms by which serotonin, working in conjunction with the gut microbiota, affects total body energy metabolism in the host. Specifically, we explore how the gut microbiota affects serotonin synthesis, release, transportation, and catabolism, and how these factors interact to regulate host energy homeostasis. The fresh perspective will help expand the understanding of serotonin in modulating energy metabolism. We also consider potential serotonin-targeting treatment options based on gut microbiota modulation in managing metabolic diseases.

## Serotonin metabolism and the impact of the gut microbiota

### Source and synthesis

**Figure 1:**
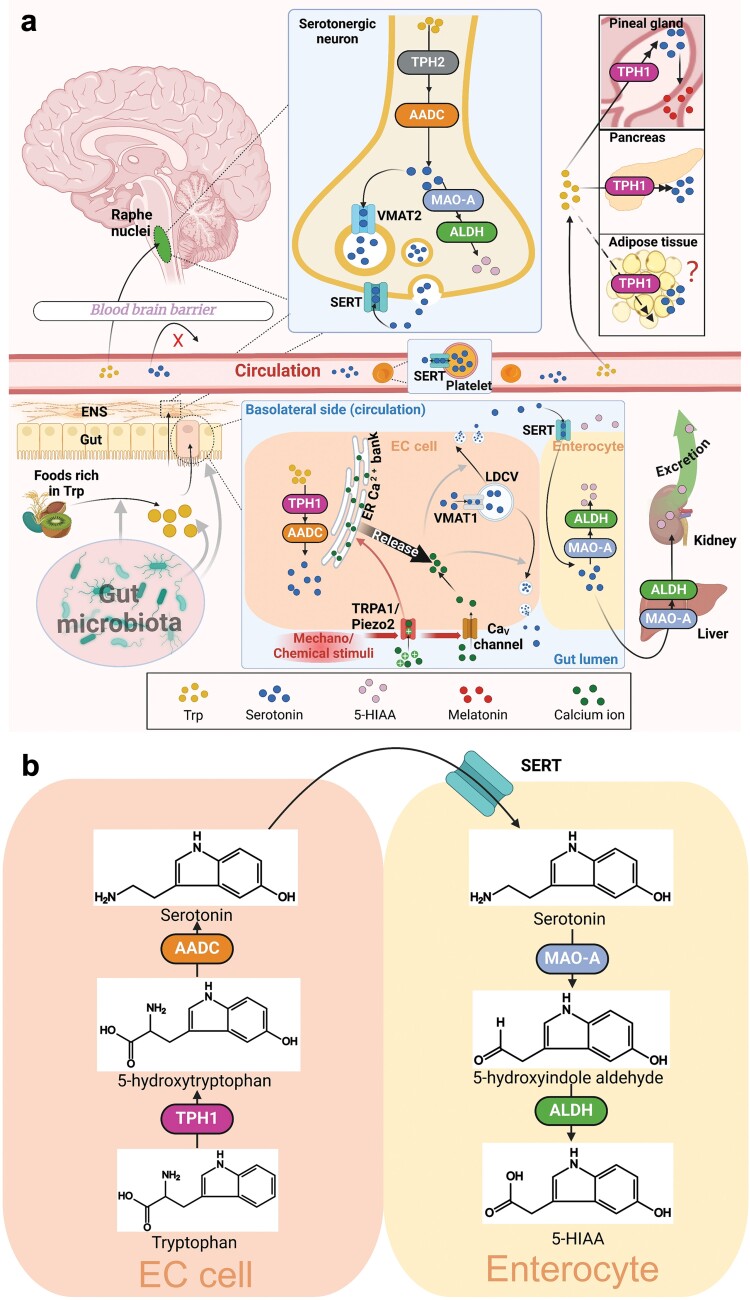
Schematic representation of the metabolic pathways of serotonin. (a) Serotonin metabolism in a variety of organs. Trp is converted into 5-HTP by TPH, mainly subtype TPH1 in EC cells of the gut and TPH2 in serotonergic neurons of the CNS and ENS, respectively. Then 5-HTP is decarboxylated to serotonin (5-HT) by AADC. In EC cells, once produced, serotonin is transported via VMAT1 into LDCVs. Upon stimulation of mechano force sensors (e.g. Piezo2) and chemical sensors (e.g. TRPA1), an electric potential difference produced by nonspecific cation entry initiates massive Ca^2+^ influx through the Ca_V_ channel, thereby eliciting ER Ca^2+^ release. Accumulation of Ca^2+^ in the cytoplasm triggers the release of serotonin. Enterocytes take up surplus serotonin via SERT and degrade part of serotonin into 5-hydroxyindoleacetic acid (5-HIAA) by mitochondrial enzyme MAO-A and ALDH. The remainder of serotonin enters the intestinal capillary plexus, where it is taken up by platelets via SERT to be stored, and to a much less extent, exist in a free form. After reaching the liver through the hepatic portal vein, part of serotonin is degraded by MAO-A, which prevents the potential harm of excess peripheral serotonin. Gut microbiota regulates the release of Trp from foods and the synthesis of serotonin in the gut. In serotonergic neurons, VMAT2 mediates the storage of serotonin within vehicles, and SERT on the surface of serotonergic neurons is involved in the reuptake of serotonin released by the neurons. Peripheral organs, such as the pineal gland, the pancreas, and potentially adipose tissue, express TPH1 to produce serotonin to fit their respective physiological functions. (b) Serotonin anabolism and catabolism within EC cells and enterocytes, detailed in chemical structures. Black solid arrows represent molecules transporting among diverse cells or biochemical reactions; grey solid arrows represent (either positive or negative) regulation of physiological processes.

In mammals, the essential amino acid tryptophan (Trp) serves as the sole source of serotonin. The metabolism of serotonin within various cells and organs is summarized in Fig. 1. Although the majority of absorbed Trp is directed into the kynurenine pathway via indoleamine 2,3-dioxygenase (IDO) in extrahepatic organs, or tryptophan 2,3-dioxygenase in the liver, culminating in NAD^+^ synthesis [19], around 3% of absorbed Trp is converted into serotonin [20]. The rate-limiting step in serotonin synthesis is the conversion of Trp into 5-hydroxytryptophan (5-HTP) by Trp hydroxylase (TPH). Subsequently, 5-HTP is decarboxylated to serotonin by the action of aromatic l-amino acid decarboxylase (AADC) [21] (Fig. 1a).

Two isoforms of TPH exist: TPH1, predominantly found in the pineal gland and peripheral tissues, and TPH2, prevalent in nervous systems (Fig. 1b). TPH1 exhibits a higher efficiency in binding and hydroxylating Trp than TPH2 [22]. TPH1 is primarily expressed in enterochromaffin (EC) cells, a type of enteroendocrine cells. EC cells account for the synthesis of at least 90% of peripheral serotonin [23]. TPH1 is also expressed at other locations to synthesize serotonin for local physiological functions. For example, in response to metabolic stress such as a sudden rise in glucose levels, the pancreatic β cells upregulate TPH1 expression to boost serotonin production, thereby enhancing the capacity to produce insulin [24]. In addition, the pineal gland expresses TPH1 to produce serotonin, as a precursor of melatonin, which is involved in maintaining circadian rhythm [25]. Peripheral serotonin is not capable of crossing the blood–brain barrier [26]. TPH2 is primarily expressed in the raphe nuclei of the brainstem which is the principal source of serotonergic neurons [27] and dominates serotonin synthesis within the CNS. In addition, TPH2 is also expressed in the enteric nervous system (ENS) for the synthesis of serotonin, which is essential for the development of ENS and for regulating gut motility [28].

### Release and transportation

Once synthesized in EC cells that exclusively express TPH1, gut serotonin is packed into large dense core vesicles (LDCVs) through the action of vesicular monoamine transporter 1 (VMAT1) (Fig. 1b) [29]. The efficiency of VMAT1 (or VMAT2 in serotonergic neurons) in sequestering serotonin is dependent on a proton gradient, which is driven by the V-type ATPase located on the LDCV membrane [30]. Upon exposure to physical or chemical stimuli, a nonspecific Ca^2+^ influx is activated, facilitating the movement of the cytoskeleton and thus assisting in the transport of LDCVs toward the basolateral membrane, resulting in the release of serotonin. Piezo2, recently identified as an EC cell mechanosensor, initiates a prolonged Ca^2+^ current upon stretching [31, 32]. While Del Rosario and colleagues have attempted to elucidate the activation process of Piezo2 through a dual G-protein interaction approach [33], the detailed molecular mechanism by which Piezo2 senses mechanical force and functions remains to be unraveled. The transient receptor potential A1 (TRPA1) ion channel, a broad-spectrum chemosensor located on the EC cell surface [34], triggers serotonin release upon chemical stimulation [35]. Notably, the Ca^2+^ currents derived from these sensor channels elicit the activation of voltage-gated calcium (Ca_v_) channels or the endoplasmic reticulum (ER) Ca^2+^ release, causing significant intracellular Ca^2+^ changes to trigger serotonin release [36, 37].

To prevent overstimulation of serotonergic receptors, extracellular serotonin released by EC cells is absorbed by neighboring enterocytes or enters the portal circulation. Enterocytes take up surplus serotonin via the serotonin transporter (SERT) (Fig. 1a and b) [38], and partially degrade serotonin by the mitochondrial enzyme monoamine oxidase A (MAO-A) [39]. Given that serotonin can readily elicit vasoconstriction and coagulation in the circulation [3, 7], a substantial portion of serotonin is sequestered from target sites by SERT-expressing platelets [40]. Although platelets express MAO-B [41], this isoform appears to have a much lower affinity for serotonin compared to MAO-A [42], allowing platelets to purely serve as vehicles for peripheral serotonin without degrading it. Although it has been noticed that under certain scenarios like inflammation and vascular damage, platelets release serotonin [43], it remains uncertain to what extent the platelet-binding serotonin regulates peripheral metabolism. Besides the majority of peripheral serotonin is stored in platelets [44], less than 5% of serotonin also circulates in a free form, which is able to interact with distal organs and regulate their metabolism, a topic that will be explored in detail in the following sections.

### Catabolism

The major part of released serotonin in EC cells and serotonergic neurons is deaminated into 5-hydroxyindole aldehyde by MAO-A, which is predominantly expressed in the gut, liver, brain, and ENS (Fig. 1a) [45]. Then, aldehyde dehydrogenase (ALDH) converts 5-hydroxyindole aldehyde into 5-hydroxyindole acetic acid (5-HIAA), the ultimate metabolite of serotonin (Fig. 1a and b). Given its stable chemical properties, Fukui *et al*. [46] proposed to replace serotonin with 5-HIAA as the primary biomarker when studying the relationship between certain phenotypes and serotonin levels. Recently, 5-HIAA was demonstrated to be a ligand for G-protein-coupled receptor GPR35 to promote the recruitment of neutrophils and play a role in inflammation resolution and bacterial clearance [47]. Besides conversion into 5-HIAA, mammals have two other routes to metabolize serotonin. As an indoleamine derivative, serotonin is a substrate of IDO, which can be converted into N-[2-(3-aminopropanoyl)-4-hydroxyphenyl]formamide [48]. In the pineal gland, arylalkyl amine N-acetyltransferase, together with hydroxyindole O-methyltransferase, transforms serotonin into melatonin [49].

### Signaling pathways

#### Serotonin receptors

Unlike proteins with unique three-dimensional structures, serotonin consists of a limited atomic composition and a relatively simple but clear structure. The wide array of serotonergic effects is mediated by diverse serotonin receptors with unique tissue specificity. To date, 13 G protein-coupled receptors, i.e. 5-HTR_1A, 1B, 1D, 1E, 1F_, 5-HTR_2A, 2B, 2C_, 5-HTR_4_, 5-HTR_5A, 5B_, 5-HTR_6_, and 5-HTR_7_, and a ligand-gated ion channel (5-HTR_3_), have been identified in mammals [50, 51]. Members of the subfamily of 5-HTR_1s_, sharing 40%−63% sequence identity in humans, are all coupled to G_αi_/G_αo_ proteins to inhibit cyclic adenosine monophosphate (cAMP) formation. Depletion or pharmacological blockade of 5-HTR_1s_-mediated signaling have been related to depressive and anxiety disorders [52]. Recent evidence has shown that 5-HTR_1A_ plays a role in appetite control, particularly alcohol and sucrose consumption [53]. 5-HTR_1B_ has also been found to play an important role in the anorectic effect induced by antimigraine drug triptan [54]. 5-HTR_2A−2C_ preferentially couple to G_q_/G_11_ to enhance intracellular inositol phosphate and Ca^2+^ concentration, with long-known roles in regulating CNS activity and muscle contraction [55]. In addition, hepatic 5-HTR_2A_ and 5-HTR_2B_ signaling might contribute to hepatic fibroblast growth factor-21 (FGF21) production and the pathophysiological mechanisms of type 2 diabetes (T2D) and metabolic dysfunction-associated fatty liver disease (MAFLD) [56]. 5-HTR_2C_ is widely expressed in the hypothalamic regions that control feeding behavior and energy expenditure, thus activation of 5-HTR_2C_ affects energy homeostasis [57]. Mice with a genetic mutation of 5-HTR_2C_ display hyperphagia prior to hyperinsulinemia, insulin resistance, impaired glucose tolerance, and weight gain [58]. 5-HTR_5s_ are G_i_/G_o_ coupled receptors and transduce signals via inhibiting adenylate cyclase activity, consequently decreasing cAMP. Activation of 5-HTR_5s_ exerts an anti-migraine effect and improves sleep [50]. On the contrary, 5-HTR_4_, 5-HTR_6_, and 5-HTR_7_ receptors couple with G_s_ receptors to preferentially trigger a second messenger cascade mediated by protein kinase A to increase cAMP. As an exception, 5-HTR_3_ facilitates a fast postsynaptic excitatory response via the non-selective influx of Na^+^ and Ca^2+^, and over-activation of 5-HTR_3_ causes vomiting, pain, schizophrenia, and intestinal motility abnormalities [59, 60]. The serotonergic system continually regulates host metabolism through the multiplicity of serotonin receptors (ionotropic and metabotropic), which possess both a fast and precise communication modality and a broader and slower way to regulate metabolic processes. Moreover, the high spatiotemporal variability of their expression is closely associated with the specificity of the serotonin effects [61].

#### Intracellular post-translational modification

Within the cytoplasm, serotonin takes part in post-translational modification of proteins, i.e. “serotonylation”, to impact molecular signaling by these proteins. Serotonylation of intracellular proteins regulates various physiological processes depending on the location within the body, such as platelet aggregation, insulin release, muscle contraction, and neuron differentiation. Many proteins, including small guanosine triphosphatases (GTPases) (Rab3a, Rab27a, Rab4, Ras homology family member A [RhoA], Ras-related C3 botulinum toxin substrate 1 [Rac1], and cell division cycle 42 [Cdc42]), cytoskeletal proteins (myosin heavy chain, α-actin, β-actin, γ-actin, and actin-binding protein filamin A), extracellular matrix protein fibronectin, and histones have been described as substrates for serotonylation [62]. Intracellular serotonin regulates insulin secretion from pancreatic β cells through serotonylation of GTPases (Rab3a and Rab27a), and mice selectively deficient in serotonin develop diabetes [63]. Serotonylation has first been shown in platelets, with serotonylation of GTPases by transglutaminases increasing the release of coagulation effectors that induce platelet aggregation [64]. An analogous mechanism has sequentially been identified in β cells of the pancreas, where serotonylation of GTPases promotes insulin secretion [63]. In addition, serotonylation of the small GTPase Rab4 in skeletal muscle cells enhances glucose transporter type 4 (GLUT4) translocation to the cell membrane and increases glucose uptake into the cell, thus improving glucose homeostasis [65].

Not only GTPases but also histones can be serotonylated. Histone serotonylation, the first identified endogenous monoamination modification of histone, is described as a novel epigenetic regulatory mechanism [66]. Farrelly *et al*. found that serotonylation of glutamine at position five on histone H3 (H3Q5ser) promotes its interplay with the transcription factor TFIID (TATA-box-binding protein [TBP] and TBP-associated factors [TAFs]) in collaboration with adjacent histone H3 lysine 4 (H3K4) trimethylation. The dual modification facilitates locus-specific gene expression during neuron differentiation and, consequently, the development of the CNS [67]. WD repeat-containing protein 5 (WDR5) was later described as a specific “reader” for H3Q5ser, which deciphers epigenetic signals to activate neurodevelopment-related genetic programs. Mechanically, WDR5 colocalizes with H3Q5ser in the promoter regions of oncogenes in neuroblastoma cells and facilitates gene transcription to induce cell differentiation and promote tumorigenesis [68].

Furthermore, abnormal serotonylation is associated with several metabolic diseases. Because intracellular serotonin regulates insulin secretion from pancreatic β cells through serotonylation of Rab3a and Rab27a, mice selectively deficient in serotonin develop diabetes [63]. In addition, elevated fibronectin serotonylation is observed in pulmonary artery smooth muscle cells of experimental animal models of hypertension and patients with pulmonary hypertension, indicating that fibronectin serotonylation may serve as a biomarker for pulmonary hypertension [69].

In conclusion, serotonin is more than a neurotransmitter. The widely expressed and diverse serotonin receptors, as well as the recently discovered role of serotonin in protein serotonylation, contribute to revealing unknown cellular mechanisms controlled by serotonin.

### Interaction between gut microbiota and serotonin

The gut microbiota, an integral consortium of organisms that maintains a symbiotic relationship within the host’s gut, exerts various essential biological functions themselves and through their metabolites. Gut-derived serotonin plays a crucial role in bridging the interaction between the host and microorganisms, providing an effective approach to understanding the impact of gut microbiota on host physiology.

#### The impact of gut microbiota on serotonin synthesis and release

By using gnotobiotic mouse models, mechanistic insight has been gained into how gut microbiota regulates serotonin levels in the gut. As given in Table 1, gut microbiota influences serotonin synthesis within the gut lumen through at least four distinct mechanisms. Firstly, certain bacterial species, including *Corynebacterium* [70], *Streptococcus* [71], and *Escherichia coli* (*E. coli*) [72], are capable of synthesizing Trp due to their inherent expression of Trp synthase [85], which provides an auxiliary source for EC cells to synthesize serotonin. Secondly, the expression of serotonin-synthesizing genes in EC cells can be upregulated by several species, such as *Akkermansia muciniphila*, *Clostridium ramosum* (*C. ramosum*), *Bifidobacterium*, *Ruminococcus gnavus*, and *E. coli* [73–78]. Thirdly, some bacterial species can metabolize Trp to kynurenine or indole derivatives, thereby reducing Trp in the gut lumen as the substrate for serotonin synthesis [80–82, 86, 87]. Finally, *Enterococcus faecalisper se* is reported to express AADC, adding to serotonin production within the gut lumen [79].

**Table 1.** Impact of gut microbiota on serotonin metabolism.

Gut microorganism	Regulatory “target”	Serotonin metabolism
*Corynebacterium* [70]	Trp level in gut lumen	Synthesis ↑
*Streptococcus* [71]		
*Escherichia coli* [72]		
*Akkermansia muciniphila* [73]	*TPH1* expression in EC cells	Synthesis ↑
*Clostridium ramosum* [74]		
*Bifidobacterium* [75, 76]		
*Ruminococcus gnavus* [77]		
*Escherichia coli* [78]		
*Enterococcus faecalis* [79]	*AADC* in EC cells	Synthesis ↑
*Peptostreptococcus russellii* [80]	Trp consumption	Synthesis ↓
*Lactobacillus spp.* [81]		
*Edwardsiella tarda* [82]	*TRPA1* in EC cells	Release ↑
*Akkermansia muciniphila* [73]	*SERT* in enterocytes	Transportation ↑
*Saccharomyces boulardii* [83]	*SERT* in enterocytes	Transportation ↓
*Turicibacter sanguinis* [84]		

Little is known about which aspects of serotonin releases could be regulated by gut microbiota except for TRPA1. Trp metabolites produced by *Edwardsiella tarda* stimulate TRPA1 on enteroendocrine cells, resulting in the secretion of serotonin to increase intestinal motility and stimulate both vagal sensory ganglia and cholinergic enteric neurons [82].

#### The impact of gut microbiota on serotonin transportation

As for serotonin transportation from interstitial space into intestinal epithelial cells, gut microbiota plays an indispensable modulating role through multiple mechanisms. Firstly, certain gut microbes, such as *Akkermansia muciniphila*, can directly modulate SERT expression on intestinal epithelia cells via secretion of specific proteins. For instance, the outer membrane protein Amuc_1100, released by *Akkermansia muciniphila*, can activate Toll-like receptor 2 in the Caco-2 intestinal epithelial cell model, leading to the downregulation of SERT expression and an increase in extracellular serotonin levels [73]. In contrast, the supernatant of *Saccharomyces boulardii* has been found to upregulate SERT expression by enhancing the release of heparin-binding epidermal growth factor and activating the epidermal growth factor receptor [83], as a consequence inhibiting gut motility, thereby providing a potential therapeutic strategy for disorders like irritable bowel syndrome (IBS). Secondly, gut microbiota can indirectly affect SERT expression by interacting with the intestinal immune system. For instance, lipopolysaccharide could stimulate mast cells to release prostaglandin E2, which subsequently inhibits SERT expression, leading to increased intestinal serotonin levels and exacerbating the inflammatory response [38].

Moreover, certain gut bacteria have been found to harbor proteins similar to the SERT, enabling them to interact with host serotonin metabolism directly. *Turicibacter sanguinis* (*T. sanguinis*) synthesizes a protein related to the neurotransmitter sodium symporter family, sharing similarities in structure and sequence with mammalian SERT [84]. This protein allows *T. sanguinis* to transport intracellular serotonin, thereby immediately increasing serotonin levels in the intestinal lumen. Interestingly, this transport process in *T. sanguinis* can be halted by fluoxetine, a drug known for its inhibitory effect on serotonin reuptake. There is no evidence yet that gut microbiota plays a solid role in serotonin catabolism.

#### Serotonin modulates gut microbiota community

As evident from the previous section, gut microbiota thus plays a crucial role in regulating serotonin metabolism in the gut. Meanwhile, gut microbiota is also subject to be modulated by serotonin signaling. Increasing intestinal luminal serotonin enriches the relative abundance of spore-forming bacteria [84]. Moreover, serotonin levels in the gut are also associated with virulent phenotypes of specific opportunistic pathogens [88, 89]. These findings suggest the existence of serotonin pathways within gut bacteria involved in regulating bacterial growth and function.

Like serotonin exerting effects on the host through a plethora of 5-HTRs, serotonin plays equally complex roles in the microbial milieu of the gut. Aman *et al.* have reported a bacterial-specific serotonin receptor CpxA, which is a membrane-bound histidine sensor kinase [90]. Serotonin triggers the dephosphorylation of CpxA, inhibiting the activation of virulence genes overseen by the transcription factor CpxR. It especially affects those genes that pose a threat to the structural integrity of the host intestine. Although bacteria do possess a neurotransmitter sodium symporter-related protein with sequence and structural homology to host mammalian SERT [84], suggesting the possibility of serotonylation within bacteria, protein serotonylation in bacteria has not been described yet. These exciting discoveries reveal that serotonin has an important but still poorly understood role in modulating the gut microbiota community, and further research is necessary to provide insights into the underlying modulation pathways of serotonin on gut microbiota.

## Serotonin regulates energy metabolism as modulated by gut microbiota

**Figure 2:**
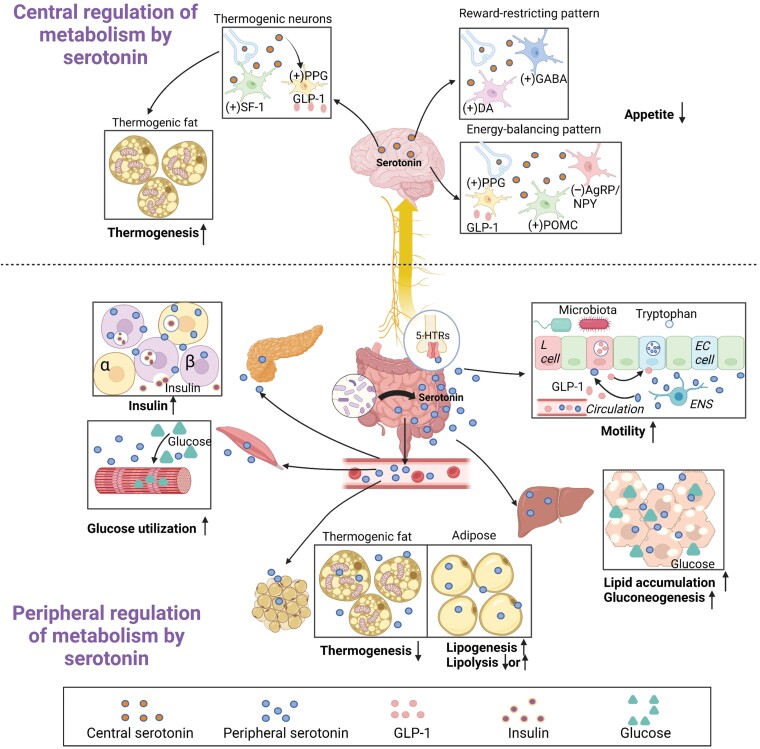
Metabolic functions of serotonin in different tissues as influenced by gut microbiota and the gut–brain axis. In the CNS, serotonin suppresses appetite via the energy-balancing pattern and the reward-restricting pattern. In addition, central serotonin promotes thermogenic fat activity potentially by increasing the activity of GLP-1 and 5-HTR-expressing steroidogenic factor-1 (SF-1) neurons. In the periphery, serotonin regulates energy homeostasis through a complex serotonergic network in key metabolic organs. Serotonin in gut lumen promotes nutrient absorption by increasing gut motility through the gut–brain axis. Serotonin in gut lumen also interacts with GLP-1 to regulate gut peristalsis. In the liver, serotonin regulates energy balance by promotion of gluconeogenesis in the fasting state and lipid accumulation in the fed state. In the pancreas, serotonin increases insulin production. In the adipose tissue, serotonin acts to store energy, by inhibiting thermogenesis in BAT and increasing lipid deposition in WAT. In the skeletal muscle, serotonin enhances glucose utilization. As peripheral serotonin is mostly derived from the gut, its influence on host metabolism is largely regulated by gut microbiota and the gut–brain axis.

Serotonin exerts metabolic effects in both the CNS and peripheral tissues through distinct serotonin receptors. Figure 2 briefly portrays the regulation of host energy metabolism by serotonin in multiple tissues, the impact of gut microbiota, and the involvement of the gut–brain axis, which will be detailed in this section.

### CNS

Serotonin within the CNS has been proven to orchestrate total body energy metabolism (Fig. 2). In animal models of obesity, baseline serotonin release from the hypothalamus was found to be lower [91]. People carrying loss-of-function variants in 5-HTR_2C_, which is primarily expressed on hypothalamic proopiomelanocortin (POMC) neurons, have hyperphagia and weight gain in childhood, leading to severe obesity [92]. A clinical trial supported that high-fat high-sugar snacking might decrease extracellular serotonin through a decreased SERT in the hypothalamic region, resulting in abnormal appetite and a higher risk of obesity [93]. Given the mounting evidence for serotonin as a key neurotransmitter in regulating energy metabolism, attempts to target serotonin signal regulation in specific brain nucleus have emerged to attenuate obesity and related comorbidities [94–98].

Decades of studies together have led to a promising proposal that central serotonin reduces food intake via two independent patterns, i.e. the energy-balancing pattern and the reward-restricting pattern [91]. Both patterns originate from serotonergic neurons in the brainstem raphe nuclei [99]. The energy-balancing pathway is activated when energy intake surpasses the body’s needs, inhibiting cerebral aspects governing food consumption. This pathway interacts primarily with metabolic state-sensing neurons such as POMC-expressing neurons and those expressing agouti-related peptide (AgRP) and neuropeptide Y (NPY) [100], which mainly produce anorexigenic effects through stimulation of 5-HTR_2C_ [101] and 5-HTR_1B_ [54], respectively. A recent study showed a serotonin 1B receptor circuit (5-HTR_1B_^AgRP^→paraventricular nucleus circuit) for appetite suppression in mice, which suggested that 5-HTR_1B_ is a potential new target in 5-HT-based weight-loss therapies. 5-HTR_1B_ and 5-HTR_2C_ act on different neural pathways for appetite suppression in mice and can be targeted for obesity treatment independently [54]. In addition, serotonin activates preproglucagon neurons in the brainstem to synthesize glucagon-like peptide 1 (GLP-1) [102], further suppressing food intake. Meanwhile, the reward-restricting pathway limits hedonic eating behavior involving the pleasure-responsible mesolimbic system, particularly dopaminergic neurons equipped with 5-HTR_2C_ [103]. Activation of 5-HTR_2C_ in these neurons diminishes binge eating and motivational food intake [104]. Serotonin-innervated gamma-aminobutyric acid (GABAergic) neurons also reduce motivational eating behavior upon 5-HTR_2C_ triggering [105]. Thus, there have been some supporting theories for the idea that overactivity of the appetite-inhibiting pathway can lead to a deficit of pleasure reward. However, obesity and depression usually exhibit high comorbidity [106], indicating the complex roles of serotonergic neurons in the modulation of motivational eating behavior.

Apart from central inhibition of appetite, there has been a rising interest in the relationship between central serotonin and the regulation of peripheral thermogenic adipose tissue [107]. Intriguingly, eliminating serotonergic neurons by diphtheria toxin impairs thermogenesis within brown adipose tissue (BAT) and beige adipose tissue, implying a vital role of central serotonin in energy expenditure [108]. 5-HTR-expressing steroidogenic factor 1 neurons in the ventral medial nucleus are proposed to be required for the modulation of thermogenesis, especially in high-fat diet (HFD) conditions [109]. In addition, pharmacological activation of central GLP-1 receptor (GLP-1R) signaling expressed by serotonergic neurons [110–112] augments the activity of thermogenic adipose tissue and enhances the utilization of circulating glucose and triglycerides [113, 114]. However, total body GLP-1R knockout (KO) mice maintain brown fat thermogenesis [114], indicating the presence of a sub-population of neurons regulating energy expenditure, and further research should focus on those extraordinary serotonergic neurons involved in appetite and thermogenesis.

The gut microbiota plays a crucial role in influencing CNS physiological homeostasis, such as mood, appetite, pain perception, and stress responses [115, 116], probably with serotonin as one of the underlying bridges [117]. Emerging evidence suggests a relationship between gut microbiota and central serotonin alterations that might impact appetite regulation [118], which is potentially attributed to changes in circulating Trp levels [119, 120]. In addition, gut-derived serotonin can stimulate 5-HTR_3_-expressing neurons in the gastrointestinal tract, which send signals to the CNS via the vagus nerve [121]. Whether gut-derived 5-HTR_3_ signals could influence appetite and energy expenditure remains unclear. Moreover, gut-derived serotonin can affect the secretion of other gut-derived hormones such as GLP-1 [122], which can cross the blood–brain barrier to exert metabolism-regulating effects in the CNS or stimulate the activity of vagal afferents similarly to serotonin [102]. This intricate interplay between gut hormones, gut microbiota, and CNS serotonin signaling provides a novel perspective on energy regulation and metabolic health, underlining the complexity of the gut–brain axis in health and disease management.

### Gut

Numerous studies suggest that peripheral serotonin also plays an indispensable role in regulating nutrient absorption and storage [123]. Gut motility is closely regulated by local serotonin, irrespective of its origin from EC cells or ENS [124, 125] (Fig. 2). Increased nutritional signaling in the lumen stimulates EC cells to secrete serotonin [126], activating the 5-HTR_3_ in afferent neurons of ENS to initiate the gut peristalsis through the gut–brain axis [121]. Meanwhile, activation of 5-HTR_4_ on EC cells evokes a more drastic release of serotonin, consolidating the intensity of serotonergic inputs to ENS, thereby maintaining gut motility [127]. TPH1/2-targeting medications have been applied to treat diseases with abnormal gastrointestinal motility [128]. In addition to gut motility, serotonin stimulates gastric emptying, as a shortage of serotonin in the gut was shown to be associated with delayed gastric emptying [129].

Besides, emerging evidence demonstrates that gut-derived serotonin interacts with other gut hormones [130]. GLP-1 is one of the most acknowledged gut hormones that improve metabolism by inducing satiety [131]. In a study using organoids, EC cells have been discovered to express GLP-1R, and GLP-1R agonism increases the secretion of serotonin [132]. Reciprocally, GLP-1-producing L cells also express 5-HTR_3/4_ to allow for regulation by serotonin [122, 133]. However, whether activation of 5-HTRs in L cells enhances GLP-1 secretion has not been described yet. Further efforts are required to verify this hypothesis and explore potentially additional interfaces between serotonin and other gut hormones.

Accumulating evidence has shown that gut microbiota fundamentally manipulates the physiology of intestinal peristalsis with serotonin as a commander. The identified microbiome–metabolome signatures for IBS, a group of diseases characterized by gut motility dysfunction, are associated with altered serotonin metabolism and unfavorable stress responses related to gastrointestinal symptoms, supporting a role of the microbiota–gut–brain circuit in the pathogenesis of IBS [134]. Indeed, depletion of gut microbiota with antibiotics impairs gut motility, which is attributed to decreased colonic serotonin [135]. Furthermore, colonization of germ-free mice with microbiota from conventional mice increases neuronal and mucosal serotonin production accompanied by elevated intestinal transit rates [136]. These studies emphasize the indispensable role of gut microbiota in regulating gut motility. In addition, in a mouse model of autism spectrum disorder, delayed intestinal transit and impaired serotonin production strongly correlated with the abundance of the *Blautia* genus of the *Clostridiales* [137]. As mentioned above in the Section “Interaction between gut microbiota and serotonin”, gut microbiota can release metabolites that regulate the expression of host serotonergic genes. For example, Zhai *et al*. proposed that phenylamine and tryptamine produced by *Ruminococcus gnavus* accelerate gastrointestinal motility and trigger IBS by increasing TPH1 expression via the stimulation of amine-associated receptor 1 [77]. Accordingly, with the orchestration of gut microbiota, serotonin maintains the homeostasis of gut motility via the gut–brain axis, thereby regulating nutrient absorption and host energy metabolism.

### Liver

As a metabolic hub, the liver is responsible for the biosynthesis and biodegradation of major energy substrates for use by peripheral tissues. Recently, serotonin has emerged as an important regulator by which the liver can adapt to environmental changes [138–140] (Fig. 2). In the context of a nutrition crisis, gluconeogenesis and glycogenolysis are triggered in hepatocytes. Under fasting conditions, gut-derived serotonin activates 5-HTR_2B_ on hepatocytes to enhance the activity of fructose-1,6-bisphosphatase and glucose-6-phosphatase (G6Pase), thus promoting gluconeogenesis, as well as to suppress hepatic glucose uptake via GLUT2, thereby maintaining circulating glucose in a coordinated manner [138]. Conversely, in diet-induced obese mice, knocking out 5-HTR_2C_ increases hepatic glucose production, indicating how serotonin regulates hepatic gluconeogenesis [139]. Fluoxetine is applied as a first-line antidepressant through increasing extracellular levels of serotonin. However, fluoxetine also induces severe metabolic disorders such as dyslipidemia and hyperglycemia, associated with downregulated hepatic G6Pase expression and enhanced transformation of glucose to lipid, which might be a serotonin-dependent mechanism facilitating hepatic steatosis [140].

Similarly, multiple studies have reported that gut-derived serotonin is closely related to lipid deposition in the liver [141–144], the core pathology of metabolic dysfunction-associated steatotic liver disease (MASLD). Total body *Tph1*-KO mice fed an HFD showed protection against hepatic steatosis [141]. It has been further shown that eliminating gut-derived serotonin by intestine-specific *Tph1*-KO markedly ameliorates hepatic steatosis [145], revealing a close relationship between gut-derived serotonin and hepatic lipid metabolism. The 5-HTR_2A_ signal in the liver has been known as the predominant transduction pathway to regulate genes like sterol regulatory element-binding protein 1c (*SREBP1c*) and peroxisome proliferator-activated receptor gamma (*PPARγ*) [145, 146], which play a crucial role in the control of hepatic lipid metabolism. Based on this understanding, gut-derived serotonin exacerbates hepatic steatosis through direct actions on liver, and gut TPH1–liver 5-HTR_2A_ axis might be a potential drug target for anti-MASLD therapies.

In addition to inducing liver steatosis, serotonin is a key player in the development of steatohepatitis. Within the catabolic pathway, serotonin is oxidized by MAO-A accompanied by generation of many free radicals, exacerbating oxidative stress in the liver [147]. Serotonin also induces the expression of inflammation factors like tumor necrosis factor α and interleukin-6, which can be prevented by the TPH1 inhibitor LP533401. In addition, during the onset of liver damage, serotonin plays a critical role in the characteristic phenotypic remodeling of hepatic stellate cells, crucially involved in collagen production, resulting in the development of hepatic fibrosis [148].

The perturbation of gut microbiota has been increasingly recognized as a causative factor in MASLD, characterized by the increase of serotonin-rising strains like *Bifidobacterium*, *E. coli*, and *Clostridium* [149]. Via the hepatic portal vein system, gut-derived serotonin facilitates the pathogenesis of MASLD [145]. Taken together, the bridging role of serotonin in maintaining liver homeostasis via the gut–liver axis is being unveiled, providing a range of potentially druggable targets.

### Pancreas

The pancreas is commonly known as a manager of overall carbohydrate homeostasis, with α cells producing glucagon to increase circulating glucose levels in the fasting or hypoglycemic states and β cells secreting insulin to decrease circulating glucose levels in the fed or hyperglycemic state. Aside from nutrition signals like glucose, the pancreas senses a group of peripheral or local signals in favor of insulin secretion [150]. Serotonin is found to co-exist and be co-released with insulin from β cells [151] (Fig. 2). The origin of this serotonin has been a subject of debate. On one hand, pancreatic β cells are regarded as amine precursor uptake and decarboxylation cells, and as such decarboxylate circulatory residual 5-HTP into serotonin [152, 153]. On the other hand, β-cell-specific elimination of *Tph1* significantly blunts serotonin production, indicating *de novo* synthesis as the primary routine to produce serotonin in the islets of Langerhans [154].

The mechanism by which serotonin regulates the activity of the pancreas is ambiguous. According to predominant evidence, serotonin in the islets exerts an insulin-upregulating effect primarily via intensifying the signaling of insulin production by β cells, promoting the proliferation of β cells, reducing the glucagon excretion by α cells, and attenuating the oxidative stress in the islets [155–159]. Most of these effects above are attributed to stimulating a cluster of 5-HTRs. In addition, upon metabolic need, GTPases are serotonylated to enhance fusion of insulin-containing granules, thereby releasing insulin [63]. To reinforce the hypoglycemic effect, local serotonin in parallel binds to the 5-HTR_1F_ of α cells to inhibit glucagon secretion [160]. Overall, pancreatic serotonin is beneficial for energy utilization by modulating the hormone secreting pattern of pancreatic cells.

Moreover, serotonin may manipulate pancreatic physiology through the gut–brain axis. Makhmutova *et al*. reported that serotonin enhances communication between pancreatic β cells and nucleus of the solitary tract in the hypothalamus via 5-HTR_3_-expressing vagal sensory axons [161]. Likewise, blockage of 5-HTR_3_ in the afferent serotonergic axons of the pancreas decreases insulin secretion. Interestingly, the pancreas excretes elastase to the gut lumen to remodel the gut microbiota into a serotonin-increasing ecosystem [162]. Thus, with the assistance of gut microbiota, serotonin regulates the pancreatic function via the gut–brain axis.

### Adipose tissue

Adipose tissue is broadly categorized into white adipose tissue (WAT) and BAT, contributing to energy storage and energy expenditure, respectively [163]. Under specific conditions, WAT can undergo a reversible transformation into a BAT-like phenotype, namely “beige” adipose tissue. This intermediate form between WAT and BAT also exhibits energy-burning capabilities akin to BAT [164]. The discovery of a comprehensive set of serotonergic genes in adipose tissue by Stunes *et al*. marked the initiation of studies into the potential link between serotonin and adipose tissue function [165]. Crane *et al*. reported that total body *Tph1*-KO protects HFD-fed mice from diet-induced weight gain, insulin resistance, and non-alcoholic fatty liver disease (NAFLD) [141]. More importantly, lacking this serotonin-producing key enzyme enhances β-adrenergic receptor/cyclic adenosine monophosphate/protein kinase A signaling and consequently increases the expression of uncoupling protein 1 (UCP-1) [141], the thermogenic marker of BAT or beige adipose tissue, implying an essential role of peripheral serotonin in the regulation of thermogenic fat activity (Fig. 2). In mice with inducible *Tph1*-KO in adipose tissue, serotonin could attenuate energy storage in WAT via 5-HTR_2A_ and enhance energy expenditure in BAT via 5-HTR_3_, respectively, underscoring the regulation of energy metabolism in different adipose tissues in a serotonin-dependent manner [166]. The similar phenotypes shown between total and adipose-tissue-specific *Tph1*-KO probably indicates the synthesis of serotonin by TPH1 in adipose tissue *in situ*, which plays an indispensable role in host metabolism (Fig. 1b). In addition, serotonin impairs browning of adipocytes *in vitro* [167], an effect that may be regulated by 5-HTR_3A_ signaling [166]. Although subcutaneous injection of the SERT inhibitor fluoxetine activated thermogenic adipose tissue activity and prevented weight gain in overfed rats [168], most studies support that enhanced central serotonergic signaling upon peripheral administration of fluoxetine increases energy expenditure [107–109, 113, 114].

Lipolysis and lipogenesis, two classical lipid metabolic processes within adipose tissue, are both regulated by serotonin. Fluoxetine administration inhibits the expression of adipose triglyceride lipase and PPAR-γ in visceral adipose tissue, accompanied with the protection from HFD-induced metabolic dysfunction [169]. While one study showed that 5-HTR_2A_ signaling suppresses lipolysis in WAT [170], another study reported that 5-HTR_2A_ signaling rather facilitates *de novo* lipogenesis in WAT [171] (Fig. 2). It seems that different subtypes of 5-HTRs exert distinct effects on lipolysis. For example, in obese mice, 5-HTR_2B_ is upregulated and found to be responsible for increasing the lipolysis in the visceral adipose tissue, which subsequently worsens insulin resistance through excess free fatty acids [172].

Moreover, gut microbiota plays a pivotal role in regulating adipose tissue function, with gut-derived serotonin being a key factor. With serotonin as an intermediate, gut microbiota can target immune cells within adipose tissue and local inflammation to improve insulin sensitivity. For instance, the prebiotic B-GOS® has been associated with the upregulation of *Bifidobacterium spp*., a demonstrated TPH1-upregulating strain, coinciding with reduced inflammation within adipose tissues [173]. Similarly, integration of dietary components like docosahexaenoic acid influences the abundance of *Lachnospiraceae* with increased circulating serotonin levels, which may subsequently improve adipose tissue inflammation and insulin sensitivity [174].

### Skeletal muscle

Serotonin also influences the metabolism of skeletal muscles (Fig. 2). Early research indicated that through the stimulation of the key glycolytic regulator phosphofructokinase (PFK), serotonin enhances glucose utilization in skeletal muscle via tyrosine phosphorylation and subsequent intracellular repositioning of PFK [175]. Analogously, a systemic review informs that long-term administration of selective serotonin reuptake inhibitors (SSRIs) raises glycogen synthase activity, a representative marker of insulin sensitivity, in human skeletal muscles [176], which is attributed to an increasing protein kinase B Ser phosphorylation. The mechanism underlying the crosstalk between serotonin and insulin-related signals might be the 5-HTR_2A_-initiating Janus kinase/signal transducer and activator of transcription pathway [177]. In addition, serotonylated GTPases promote the translocation of GLUT4 to the membrane of myocytes [65], reinforcing glucose utilization by skeletal muscles.

Dysfunction of insulin sensitivity and glucose metabolism can be caused by sarcopenia, a degenerative disorder characterized by reduced muscle mass [178]. Van Long *et al*. enumerated a cluster of circulatory biomarkers to diagnose sarcopenia in C57BL/6J mice, with decreased serotonin as a sarcopenia indicator [179]. Compared with healthy individuals, patients with sarcopenia have an abnormal gut microbiome profile characterized by the alteration of certain gut microbes correlated with host serotonin metabolism [180]. A metagenomics analysis from the Xiangya Sarcopenia Study demonstrated that the abundance of *Clostridium*, an enhancer of the expression of TPH1 in EC cells of the gut, is increased in the sarcopenia group [181]. On the other hand, transplantation of *Bifidobacterium*, another reported TPH1 expression enhancer, isolated from a weightlifting athlete to rodents improved the function of skeletal muscle including grip strength and endurance without toxicity, accompanied by normal serum lactate, urea nitrogen, and creatine kinase [182]. These seemingly contradictory results indicate the complexity of how gut microbiota regulates the metabolism of skeletal muscle, and warrant further elucidation of the exact role of gut-derived serotonin in skeletal muscle metabolism.

## Clinical application of serotonin in metabolic disorders

Beyond regulating a wide range of physiological processes, serotonin is also implicated in various neuropsychiatric disorders (e.g. depression, anxiety, schizophrenia, obsessive-compulsive disorders, addiction, attention-deficit hyperactivity disorders, and Alzheimer’s disease) [183]. In addition, it plays an important role in diseases that affect peripheral organs, including gastrointestinal disorders (e.g. IBS and inflammatory bowel disease) [184] and cardiovascular diseases (e.g. arrhythmias, hypertension, and pulmonary hypertension) [185]. A variety of drugs targeting serotonin are currently used for diverse clinical purposes. Remarkably, in recent decades, the understanding of serotonin’s role in metabolic diseases has greatly increased. A growing body of evidence shows associations of genetic variants of TPH1/2, SERT, and 5-HTRs with metabolic diseases such as obesity, T2D, and MAFLD [186–192]. In addition, increased circulating serotonin and its metabolites are associated with obesity [46], and are speculated as biomarkers for diagnosis of metabolic disorders [193]. These collective findings imply that targeting serotonin could be a potential therapeutic approach for treating metabolic diseases. Indeed, a cluster of molecules related to the serotonergic system has been reported to influence serotonin levels and host metabolism (Table 2).

**Table 2.** Drugs selectively targeting certain aspects of serotonin metabolism and signaling pathways.

Drug	Target and function	Progress in clinic	Influence on metabolism	Association with gut microbiota
PCPA	TPH inhibitor	Withdrawn due to psychiatric disturbances after listed for carcinoid syndrome	Promotes energy expenditure and increases food intake in rodents [166]	Unknown
LP-533401	TPH inhibitor	Preclinical	Promotes energy expenditure and ameliorates glucose metabolism in rodents [166]	Unknown
Citalopram	SSRI	Listed mainly for MDD	Promotes insulin sensitivity in humans [194]	Unknown
Paroxetine	SSRI	Listed mainly for MDD	Fat accumulation and glucose intolerance in rodents [195]	Unknown
Fluoxetine	SSRI	Listed mainly for MDD	1. Reduces appetite and loses weight in short-term use in rodents and human [169, 196, 197] 2. Leads to overweight in long-term use in rodents and humans [198, 199]	1. Alters gut microbiota composition [84] 2. Decreases *Firmicutes*/*Bacteroidetes* ratio [200]
Escitalopram	SSRI	Listed mainly for MDD	Reduces food intake in humans [201]	Changes gut microbiota to produce more I3PA [202, 203]
Fluvoxamine	SSRI	Listed mainly for MDD	Fat accumulation in liver in rodents [204]	Unknown
Harmine	Selective MAO-A inhibitor	Preclinical	Improved β cell proliferation in rodents (probably untargeted effect) [205]	A metabolite of gut microbiota [206, 207]
NLX-101	Selective 5-HTR_1A_ agonist	Phase Ⅰ clinical trial for several mental diseases	Reduces consumption of high-sugar food in rodents [53]	Unknown
TCB-2	Selective 5-HTR_2A_ agonist	Preclinical	Suppresses lipolysis in primary white adipocytes of rodents [170]	Unknown
SB-204741	Selective 5-HTR_2B_ antagonist	Preclinical	1. Suppresses lipolysis in primary white adipocytes of rodents [172] 2. Prevents glucose stimulated insulin secretion in rodent-derived pancreatic β-cell line MIN6 [208]	Unknown
Locaserin	Selective 5-HTR_2C_ agonist	Withdrawn due to cancer risk after listed for obesity	Reduces body weight and HbA_1c_ in individuals with overweight [209]	Increases *Firmicutes/Bacteroidetes* ratio [210]
m-CPBG	Selective 5-HTR_3_ agonist	Preclinical	1. Impairs the thermogenic activity of immortalized brown adipocytes of rodents induced by β3AR agonist [166] 2. Elevates rectal temperature and reduces appetite in rodents [211]	Unknown
Ondansetron	Selective 5-HTR_3_ antagonist	Listed for vomiting caused by chemotherapy and radiation therapy	Enhances the thermogenic activity of immortalized brown adipocytes of rodents induced by β3AR agonist [166]	Unknown

MDD, major depressive disorder.

Considering serotonin’s peripheral effects on inhibiting thermogenesis in BAT and promoting lipid accumulation in WAT and liver, reducing peripheral serotonin levels could be an attractive attempt to ameliorate metabolic disorders. Inhibition of TPH, the rate-limiting enzyme for serotonin biosynthesis, in EC cells of the gut and the brain effectively decreases serotonin levels. The first-generation TPH inhibitors are phenylalanine analogues. Among these, p-chlorophenyl alanine (Fenclonine, PCPA) was once reported in a clinical trial to treat mental dysfunctions [212], but ended because of its side effects caused by unwanted flow into other amino acid channels [213]. PCPA inhibits serotonin synthesis by occupying Trp-binding site of TPH [214]. Oh *et al*. found that intraperitoneal administration of PCPA mitigates body weight gain by promoting adipose tissue thermogenesis in mice [166]. Nonetheless, PCPA can cross the blood–brain barrier and increase food intake [166]. The second-generation TPH inhibitors exclusively act on peripheral tissues [215]. LP-533401 has ameliorated adipose tissue weight and improved glucose metabolism in rodents regardless of oral [166] or intraperitoneal [141] administration. In addition, Chajadine *et al*. showed that inhibition of gut-derived serotonin production by LP-533401 could alleviate inflammation and atherosclerosis development in mice [216]. Interestingly, a clinical TPH inhibitor Telotristat etiprate (LX-1032 and LX-1606) has been approved to treat carcinoid syndrome diarrhea [217] and reduce intestinal inflammation [218], but has not been evaluated in metabolic disorders yet.

Inhibitors for SERT and MAO-A are to prevent serotonin from degradation and enlarge its bio-effects. Medications that reduce serotonin absorption by SERT are known as SSRIs, which are commonly used as antidepressant drugs. The first globally marketed SSRI was fluoxetine in the 1990s. Since then, the effects of fluoxetine on appetite and body weight have been observed. Heisler *et al*. found that fluoxetine reduces caloric intake and body weight in a dose- and time-dependent manner in rats [94]. In line, Chiu *et al*. demonstrated that fluoxetine decreases fat accumulation, probably attributed to decreased food intake [169]. Some small short-term clinical trials [194, 196, 197, 201] showed that not only fluoxetine but also some other new class of SSRIs like citalopram and escitalopram ameliorate binge eating behavior and induce body weight loss [219]. Despite these short-term benefits, increasing clinical research [198, 220–223] has demonstrated that long-term SSRI use is associated with metabolic risks. Notably, Gafoor *et al*. analyzed a cohort for over 10 years and found that long-term use of SSRIs has a high risk of weight gain [198]. Short-term SSRI use induces weight loss seemingly due to temporary appetite suppression and gastrointestinal serotonergic effects. However, with prolonged use, neuroendocrine adaptations and alterations in serotonergic regulation of appetite may lead to weight gain, as homeostatic mechanisms gradually adjust metabolic adaption over time. Another side effect of SSRIs is the retention of redundant serotonin in the liver and adipose tissue, leading to glucose intolerance and fat accumulation [195, 204]. Given the complex results of SSRIs on metabolism, issues remain to be solved before applying SSRIs to treat metabolic disorders.

MAO inhibitors, known as antidepressants, were developed in 1950s. Phenelzine, pargyline, mebanazine, and harmine were all reported to exert glucose-lowering effects via increasing insulin secretion in β cells [205, 224, 225]. Moreover, phenelzine, pargyline, and selegiline were shown to markedly alleviate fat deposition in rodents fed a high-caloric diet [226–229]. However, food intake was barely documented in these studies, leaving the effects of MAO inhibitors on appetite unknown. Because MAO inhibitors target not only serotonin but also other monoamines like catecholamine hormones [230], one should be cautious to apply them to treat metabolic disorders.

Since the 1990s, multiple agonists and antagonists for each serotonin receptor have been developed. However, very few are with high specificity. In the CNS, 5-HTR_2C_ takes command of negative appetite regulation [92], assisted by 5-HTR_1A_ [231]. Most 5-HTR_2C_ (ant)agonists share affinity with 5-HTR_2A_ and 5-HTR_2B_ because of the structure similarity among these three subtypes [232]. Lorcaserin, a high-selective 5-HTR_2C_ agonist that inhibits appetite [233], could alleviate obesity and diabetes in humans [209] with cardiovascular safety [97]. However, it has been withdrawn because of increasing the risk of cancer [234]. No other 5-HT_2C_ agonists have been evaluated in humans for treating metabolic disorders yet. Recently, Beecher *et al*. demonstrated that a selective 5-HTR_1A_ heteroreceptor agonist NLX-101 (a.k.a. F15599; 3-chloro-4-fluorophenyl-[4-fluoro-4-[[(5-methylpyrimidin-2-ylmethyl)amino]methyl]piperidin- 1-yl]methanone) reduces sucrose consumption in mice fed a high-sugar diet [53], suggesting that targeting 5-HTR_1A_ autoreceptors might represent a promising therapeutic strategy to combat obesity. In addition to those receptors that are predominantly present in the CNS, targeting peripheral 5-HTRs is also a valuable strategy for treating metabolic diseases. Given the fact that adipocyte-specific knockdown of 5-HTR_2A_ decreased lipogenesis, and adipocyte-specific knockdown of 5-HTR_2B_ decreased lipolysis, respectively [171, 172], 5-HTR_2A_ and 5-HTR_2B_ counteract each other to maintain lipid hemostasis in adipose tissues. Correspondingly, administration of a selective 5-HTR_2A_ agonist 4-bromo-3,6-dimethoxybenzocyclobuten-1-yl)methylamine hydrobromide (TCB-2) suppresses lipolysis in primary adipocytes [170], and a selective 5-HTR_2B_ antagonist SB-204741 reverses the phosphorylation of hormone-sensitive lipase *in vitro*, suggesting decreased lipolysis. In addition, SB-204741 prevents glucose-stimulated insulin secretion in pancreatic β-cell line MIN6 [208]. Ondansetron, a selective 5-HTR_3_ antagonist, could increase the thermogenic activity in immortalized brown adipocytes, while this effect is mitigated by the 5-HTR_3_ agonist meta-chlorophenylbiguanide (m-CPBG) [166]. Notably, it was shown that injecting m-CPBG into rats increases body temperature and reduces appetite [211], suggesting the complex effects of 5-HTR_3_ signaling on whole-body metabolism. Because 5-HTR_3_ is highly expressed in vagal-nerve-dominating organs such as the pancreas and intestine, it allows this ligand-gated cation channel to transmit signals to the CNS [161, 235].

Of particular importance is the gut microbiota’s interaction with medicines that target the serotonergic system to regulate host energy metabolism. For instance, one of the SSRIs, fluoxetine, was shown to increase fecal serotonin level, accompanied by an alteration in gut microbiota composition [236]. For other SSRIs, Wang *et al*. illustrated treating depressed patients with SSRI escitalopram shifted gut microbiota to produce more indole-3-propionic acid (I3PA) [200], while excess I3PA could lead to obesity and hepatic fibrosis [202, 203]. Plant-derived harmine can be fermented by gut microbes and display selective MAO-A inhibiting effects [206]. In parallel with the metabolic benefits of several selective MAO-A inhibitors, Zhu *et al*. [207] reported that harmine is negatively associated with body weight, fat mass weight, and plasma triglycerides. Song *et al*. [210] found that the metabolic benefits of the selective 5-HTR_2C_ agonist lorcaserin are accompanied by an increase in the *Firmicutes*/*Bacteroidetes* ratio. Taken together, gut microbiota plays crucial roles not only in host metabolism but also in regulating serotonergic system and impacting the efficiency of medicines to treat metabolic diseases.

## Conclusion and perspective

As one of the oldest neurotransmitters in evolutionary terms, serotonin has a simple chemical structure and a wide range of physiological functions. Emerging evidence suggests that serotonin has a profound and distinguished influence on appetite, energy expenditure, lipid and glucose metabolism, and overall metabolic health simultaneously, apart from its classical role in maintaining mental health. Strikingly, the effects of serotonin on metabolism homeostasis rely on the complexity of the serotonergic system, which encompasses synthesis, storage, release, transport, catabolism, and receptor signaling. More importantly, the gut microbiota strongly influences the serotonergic system and complex web of interactions among serotonin and multiple organs. Although the detailed molecular mechanisms by which serotonin contributes to or ameliorates metabolic diseases are still rather obscure, this review provides a new perspective to understand, discover, and elucidate the function of serotonergic system in a spectrum of physiological and pathological conditions. By combining serotonin modulating strategies and intervention to manipulate gut microbiota, innovative treatment tools for precision medicine of metabolic disorders can be developed. In conclusion, future research should focus on dissecting mechanisms underlying the effects of serotonin on metabolism directly (locally) and indirectly (systemically) and searching for new targets related with the serotonergic system, to lead to a new era in metabolic health management.
